# Establishing the Swiss Spinal Tumor Registry (Swiss-STR): a prospective observation of surgical treatment patterns and long-term outcomes in patients with primary and metastatic spinal tumors

**DOI:** 10.3389/fsurg.2023.1222595

**Published:** 2023-07-28

**Authors:** Edin Nevzati, Nicolas Poletti, Alexander Spiessberger, Sabrina Bäbler, Gabriela Studer, Christian Riklin, Joachim Diebold, Grégoire P. Chatain, Michael Finn, Jens-Peter Witt, Manuel Moser, Luigi Mariani

**Affiliations:** ^1^ Department of Neurosurgery, Cantonal Hospital of Lucerne, Lucerne, Switzerland; ^2^ Department of Spine Surgery, Cantonal Hospital of Lucerne, Lucerne, Switzerland; ^3^ Department of Neurosurgery, University Hospital of Basel, Basel, Switzerland; ^4^ Department of Neurosurgery, Cleveland Clinic, Cleveland, OH, United States; ^5^ Department of Radiation-Oncology, Cantonal Hospital of Lucerne, Lucerne, Switzerland; ^6^ Department of Oncology, Cantonal Hospital of Lucerne, Lucerne, Switzerland; ^7^ Department of Pathology, Cantonal Hospital of Lucerne, Lucerne, Switzerland; ^8^ Department of Neurosurgery, University of Colorado Anschutz Medical Campus School of Medicine, Auror, CO, United States

**Keywords:** cancer, spinal tumor, spinal metastatic disease, spine surgery, radiation oncology, registry, quality of life

## Abstract

**Background:**

Tumors of the vertebral column consist of primary spinal tumors and malignancies metastasizing to the spine. Although primary spine tumors are rare, metastases to the spine have gradually increased over past decades because of aging populations and improved survival for various cancer subtypes achieved by advances in cancer therapy. Metastases to the vertebral column occur in up to 70% of cancer patients, with 10% of patients demonstrating epidural spinal cord compression. Therefore, many cancer patients may face spinal surgical intervention during their chronic illness; such interventions range from simple cement augmentation over decompression of neural elements to extended instrumentation or spinal reconstruction. However, precise surgical treatment guidelines do not exist, likely due to the lack of robust, long-term clinical outcomes data and the overall heterogeneous nature of spinal tumors. Objectives of launching the Swiss Spinal Tumor Registry (Swiss-STR) are to collect and analyze high-quality, prospective, observational data on treatment patterns, clinical outcomes, and health-related quality of life (HRQoL) in adult patients undergoing spinal tumor surgery. This narrative review discusses our rationale and process of establishing this spinal cancer registry.

**Methods:**

A REDCap-based registry was created for the standardized collection of clinical, radiographic, surgical, histological, radio-oncologial and oncological variables, as well as patient-reported outcome measures (PROMs).

**Discussion:**

We propose that the Swiss-STR will inform on the effectiveness of current practices in spinal oncology and their impact on patient outcomes. Furthermore, the registry will enable better categorization of the various clinical presentations of spinal tumors, thereby facilitating treatment recommendations, defining the socio-economic burden on the healthcare system, and improving the quality of care. In cases of rare tumors, the multi-center data pooling will fill significant data gaps to yield better understanding of these entities. Finally, our two-step approach first implements a high-quality registry with efficient electronic data capture strategies across hospital sites in Switzerland, and second follows with potential to expand internationally, thus fostering future international scientific collaboration to further push the envelope in cancer research.

## Introduction

### Overview

**Figure 1:**
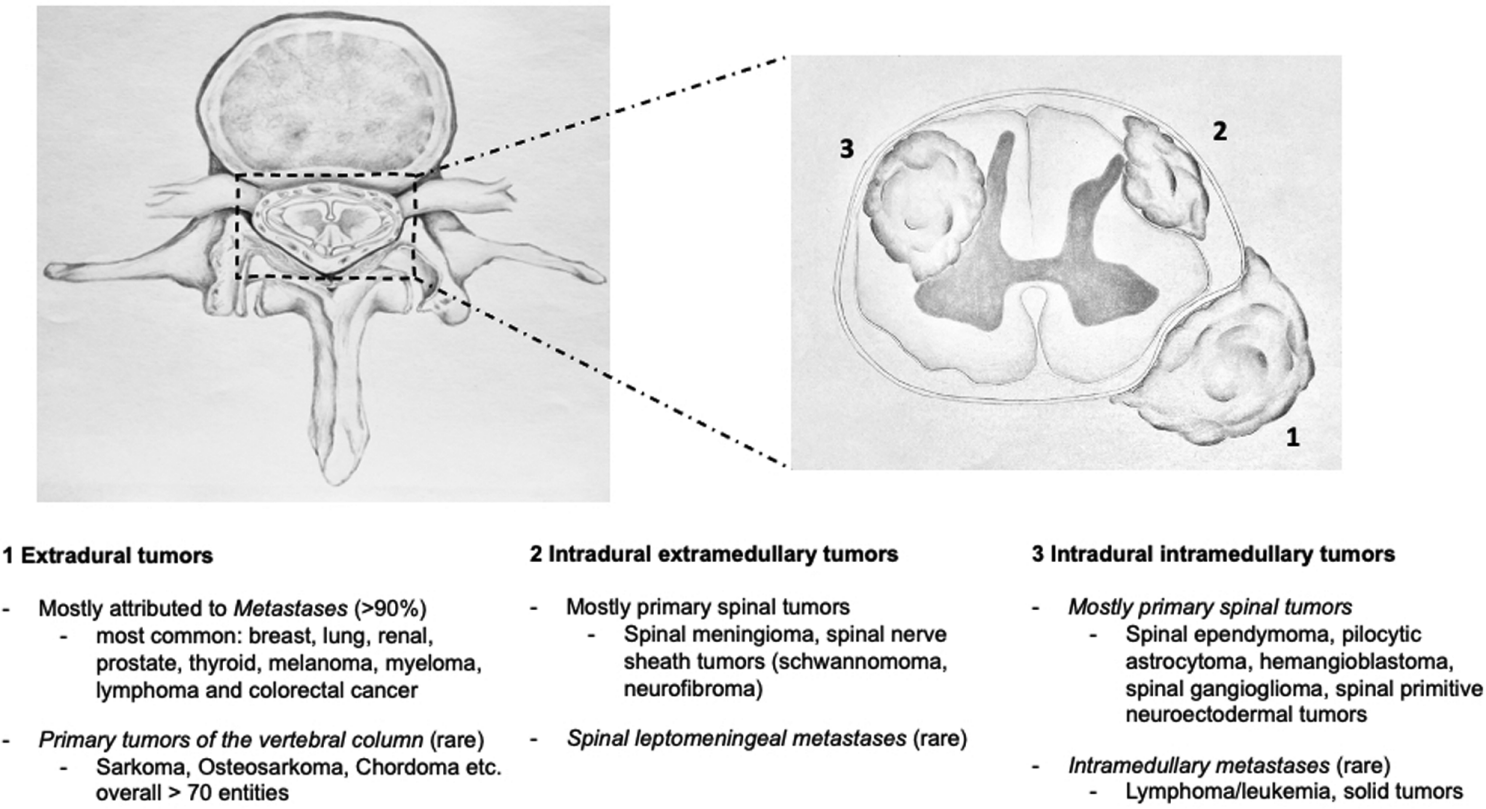
Spinal tumors can occur in any part of the spine. This figure depicts a classification system of spinal tumors relative to the spinal canal and the most frequent entities.

Tumors of the vertebral column consist of primary spinal tumors and secondary malignancies metastasizing to the spine. Primary tumors arise from the spinal cord, cauda equina, nerve roots, and spinal meninges. The surrounding vertebrae and their enveloping soft tissues can also give rise to primary tumors of the vertebral column. Compared with secondary malignancies to the spine, primary spinal tumors are relatively rare (1–3) and account for less than 10% of all vertebral column tumors (4) (Figure 1). Considering the aging population along with improved survival for multiple cancer subtypes through ongoing advances in cancer therapy, the rising incidence of metastatic spine disease is predicted to continue (5).

The spine is the most common site for bone metastases (6). Up to 70% of cancer patients develop spinal metastases during the course of their disease (7), with pain as the most frequent initial symptom (8). However, epidural tumor extension can also lead to neural compression and potential neurological deficits, as up to 10% of cancer patients develop metastatic spinal cord compression (7). Furthermore, tumors affecting the osseous spine can significantly impair mechanical integrity, potentially leading to spinal instability. The treatment of metastatic spinal lesions is considered palliative and aims to alleviate pain, maintain or improve neurological function, and restore mechanical stability. For patients with oligometastatic disease, the therapeutic intention, which is usually locally curative, requires challenging surgical or radiotherapeutical treatments. Treatment decision-making is based on neurologic, mechanical, oncologic, and systemic considerations by a multidisciplinary team effort of surgeons, radiation and medical oncologists, pain specialists, and interventional radiologists (9, 10).

As modalities available to treat metastatic spine disease evolve, the guiding principle is to improve quality of life and preserve ambulation in affected patients. However, treatment modalities vary not only globally but among different institutions within individual countries. Therefore, the potential to improve patient outcome and reduce the disease-associated socio-economic burden remains exceptionally high. Unlike metastases, primary spinal tumors represent uncommon lesions that affect a minority of the population. Nonetheless, these tumors can cause significant morbidity due to limb dysfunction and increased mortality. Consequently, the rarity of these tumors can significantly constrain the extent of research, treatment decision, and health care planning to affect patient outcomes (1).

### Role of the spine surgeon

Surgical treatment in spinal oncology has gained significant attention for its ability to reduce pain, maintain or improve neurological function, restore mechanical stability, and even improve life expectancy in patients with metastatic lesions (11–14). The implementation of stereotactic body radiation therapy (SBRT) has lessened the invasiveness of surgical treatment (9). Patients with a mechanically stable vertebral body formerly underwent radiation as local single modality treatment with palliative or locally curative (i.e., ablative) intent. SBRT has improved treatment by safely delivering high doses of radiation to the tumor while minimizing radiation dose to the surrounding organs at risk, such as the spinal cord (15, 16).

Despite recent eminent progress in the field of spinal oncology, surgery in spinal malignancies remains palliative. The surgical treatment plan not only relies on neurological and biomechanical aspects but considers the systemic tumor effects and patient's expectations. It is well accepted that surgery improves quality of life (QoL) in metastatic spine disease, even for patients whose life expectancy is <3 months (17). However, the tolerance for complications is relatively low in this patient population awaiting adjuvant therapy. For example, a surgical side infection can significantly reduce the life expectancy of affected individuals (18). Therefore, a tailored surgical approach to lower perioperative morbidity is of outmost importance for this population. The broad armamentarium in spine surgery has launched healthcare providers into uncharted terrain. Therefore, more often, the spine surgeon must weigh to what extent spine surgery can be applied in a palliative situation.

## Methods

The objective of the Swiss Spine Tumor Registry (Swiss-STR) is to obtain prospectively collected data in adult patients (age >18 years) undergoing surgery for tumors affecting the spine, irrespective of suffering from metastatic spine disease or primary spinal tumors. The cornerstone parameters to define crucial characteristics in spinal oncology include patient-reported outcome measures (PROMs) using established health-related quality of life (HRQoL) questionnaires, assessment of neurological performance, radiographic assessment to evaluate mechanical stability of the spine, oncological assessment, and tumor tissue (and whole blood) preservation. Standardized measuring tools of Swiss-STR are summarized (Table 1). Furthermore, the establishment of a biobank forms a main component of personalized medicine that allows for proteomic, metabolomic and epigenetic testing: these components form the three main pillars in cancer research (19).

**Table 1:** Standardized measuring tools.

Parameter	Measurement tool
Health-related quality of life	Spine Oncology Study Group Outcome Questionnaire (SOSGOQ2.0) EuroQol-5 Dimensions (EQ-5D) Pain Visual Analog Scale (VAS) Pain medication use, Morphine Milligram Equivalents per day (MME)
Neurological performance	Neurological examination Timed Up & Go (TUG) Test Epidural Spinal Cord Compression Scale (ESCC) Karnofsky Performance Status
Spinal Stability	Spinal Instability Neoplastic Score (SINS) Weight bearing whole spine static imaging (EOS)
Oncological assessment	TNM(G) Status Tumor genetic profiling Oncological treatment Radio-Oncological treatment Surgical Treatment
Tumor tissue	Stored in Biobank

Our study protocol was reviewed by the corresponding state institutional review board (Ethical Committee of Northwestern and Central Switzerland) and deemed not to require Institutional Review Board approval, as the registry exclusively collects encrypted prospective patient data. Written informed consent by patients will be obtained.

### Health-related quality of life

Various assessments have been developed to quantify how spinal tumors impact the patient's psychological, socio-economical, and medical condition. The HRQoL assessment aims to record how the disease and treatment affects patients' overall function and well being. Although general questionnaires have been used to assess metrics in patients with spinal tumors, a disease-specific survey was first introduced as the Spine Oncology Study Group Outcome Questionnaire (SOSG-OQ2.0) to measure QoL in patients with spinal metastases (20–22). This 27-question survey covers six dimensions that include pain, mental health, social interaction, bowel and bladder function, physical activity and neurological function. To date, validated translations of the English SOSG-OQ2.0 are available in Italian, Thai, Chinese, Dutch, and German (23–27); the latter is used for our registry. The EuroQol-5 Dimensions Questionnaire (EQ-5D) is a well known and widely used tool of 5 questions covering 5 dimensions, namely ambulation, autonomy, activities of daily life, pain, and psychological situation. It has a high shared variance with the SOSG-OQ (21). A validated German translation of the EQ-5D will be used for our registry (28). As part of this health-related QoL assessment, the pain numeric rating scale (NRS) will be scored and pain medication, particularly opioid use, will be monitored over time.

### Neurological performance and compression of neural structures

Neurological impairment and the presence of spinal cord and/or radicular compression play a key role in treatment decision-making. In their systematic review, Nguyen et al. (29) assessed ambulation in patients with spinal metastases and found there was no standard measure of ambulation for this population. Rather, most studies included classified patients as either *ambulatory* or *non-ambulatory*, and a few designated *ambulatory aided*. Of the 12 prospective studies, 11 studies used direct observation to grade ambulation though the method of determination was rarely described.

Given the significance of ambulation on QoL and overall morbidity, quantification by standardized tests rather than observation (observational “in or out” criteria) or questionnaires is needed. For this measure, the widely used Timed Up & Go (TUG) test will be applied to assess ambulatory function (30). Evaluated in general oncologic patients undergoing elective surgery, the TUG better predicted postoperative morbidity than the American Society of Anaesthesiologists (ASA) Score (31). Although it has also been evaluated in degenerative spine disease (32), no studies have thus far evaluated TUG exclusively in spinal oncologic patients.

Additionally, the Karnofsky Performance Status will be assessed as a basic performance status in oncological patients (33). Besides assessing obvious neurological deficits in the clinical assessment, an anatomical, MRI-based 6-point grading system will delineate the degree of spinal cord compression. The epidural spinal cord compression (ESCC) scale was introduced in 2010 by Bilsky et al. The rationale behind this grading system was to provide a reliable tool for assessment of spinal cord compression, and thus consistency in reporting outcomes of treated spinal tumors (34).

### Spinal stability

Mechanical instability as a result of a neoplastic process can lead to an indication for surgery, independently from the presence or absence of neural compression or estimated response to systemic and radiation treatment (35). To facilitate assessment of stability and unite reporting among health care providers, the Spinal Instability Neoplastic Score (SINS), introduced in 2010 (36), was based on conventional radiography and computed tomography (CT) scans. The SINS is calculated from 6 components and classifies lesions as stable, potentially unstable, and unstable. Although the role of sagittal imbalance has been analyzed extensively in deformity surgery (37), it has not yet in spinal tumors (38). Ideal sagittal alignment of the spine improves biomechanical efficiency and reduces energy expenditure on accessory muscles to stay erect. To analyze sagittal balance in tumor patients, data from a weight-bearing whole-spine-posture imaging is included in the registry.

In summary, all patients recruited for Swiss-STR will have baseline spinal CT, contrast-enhanced MRI, and x-ray before surgery. A postoperative MRI scan will be conducted to assess tumorreesction/resdidual tumor burden, and hardware placement will be assessed on postoperative x-ray.

### Systemic tumor assessment

The most common tumors metastasizing to the spine are prostate and lung cancer in males, and breast cancer and lung cancer in females (39, 40). Overall survival has improved significantly over the last two decades for all three pathologies (41, 42). A growing number of cancer-specific treatment options have emerged for metastatic cancer (43). Because of longer survival for cancer patients, demographics have shifted and many survivors are now older than 65. This comes with more age-related morbidity and thus more complex cases (44). A new paradigm emerged as metastatic cancer can be a chronic disease rather than a lethal diagnosis.

Scores currently available that predict survival, when deciding whether or not to operate on a patient, originated in the 1990s and early 2000s, like the Bauer-, Tokuhashi- and Tomita-Score (1995, 1990 and 2001) (13, 45, 46). Although revised scores were developed, their performance is poor because treatment options, demographics, and survival differ substantially from the time these scores were created (47). In their 2016 systematic review, Zoccali et al. found the accuracy of the Tokuhashi-Score in predicting survival was only 63%, with a decreasing trend over time (48).

This highlights the importance of developing new scoring systems with up-to-date patient data. Increasing evidence has emerged that preoperative survival estimation should rely on flexible model techniques, such as machine learning (49). Emerging new treatment options in metastatic cancer are targeted therapies and immunotherapies. An increasing number of molecular subtypes and thus potential targets exist for cancer therapy (50). In their review, Yuan et al. identified 356 clinical studies worldwide in 2019 investigating treatment of non-small cell lung cancer with combinations of immunotherapy (51). Given that the problems with targeted therapies are acquired resistances (52), there is a need to analyze a tumor's molecular evolution when metastasizing to the spine.

To evaluate the important factors affecting the outcome in our spinal tumor patients, we will document spine-related symptoms, radiological features, and treatment of the spine and also oncological, radio-oncological, and surgical details of the primary tumor, together with secondary diagnosis and general health status. The 12 most common secondary diseases and risk factors with corresponding important laboratory values and medication will be documented Supplementary Table S1. To assess nutrition status, we will collect the nutrition risk score and the Scored Patient-Generated Subjective Global Assessment (SG-SGA)- a detailed and well evaluated questionnaire to assess nutrition status in oncologic patients (53, 54). Cachexia and sarcopenia are considered important risk factors for complications not only when undergoing surgery but also radio- or chemotherapy (55). Several recent studies have shown a significant correlation between sarcopenia and postoperative complications and mortality in spinal tumor patients undergoing surgery (56, 57). The sarcopenia index of the psoas will be measured in preoperative CT scans at the L3 level as previously described (56).

### Clinical database management

Spinal oncology research based on the registry will involve collaborations with many centers in diverse locations relying on electronic networks that enable submission, analysis, and sharing of data. However, paramount importance is to secure data collection, storage, and export for any multi-center research study. Swiss-STR will use the Research Electronic Data Capture (REDCap®, Vanderbilt University) software application system that has successfully supported translational research projects between academic centers (58).

The comprehensive data collection forming the registry will be time consuming and rely on dedicated investigators and study nurses. However, extraction of data directly from electronic health record systems into REDCap has a great potential to facilitate and optimize the maintenance of the registry. After first establishing Swiss-STR at the Cantonal Hospital of Lucerne in Switzerland, patient recruitment began on January 1, 2023. Our next goal is to involve other public spine centers with expertise in spinal oncology on a national and international scale. The value of the registry heavily relies on the data quality and quality control procedures. Evaluation of data quality in tumor registries is expressed by the dimensions of comparability, completeness, validity, and timelines (59). Although these attributes primarily evolved for large scale/national registries, the principles can also be applied to institutional-based registries.

The data collected in the Swiss-STR adheres to agreed international guidelines in the diagnosis and treatment of spinal oncological disease, thereby ensuring comparability of entered parameters. All surgically-treated spinal oncological patients will be included in participating centers by a dedicated, clinical practice of approved staff who can complete prospective data collection. The validity/accuracy of data collection is defined as the proportion of cases in a dataset with a given characteristic which truly have the attribute (59). Participating centers will hold periodic scientific exchanges to assess the growth of the clinical database and an independent expert data-monitoring committee will assess data quality in timely defined audits, using reabstracting and recoding methods (60). The term *timeline* relates to the rapidity in collecting, processing, and reporting complete data out of the registry (61). As the registry is an institutional-driven prospective-data-gathering entity, data will primarily flow into data capture system to enable timely available, efficient data processing. Annual reports will provide basic epidemiological data while research projects can be coordinated and specific data can be allocated to address a scientific question.

## Discussion

In this review, we present our rationale for the need and process of establishing a spinal cancer registry called the Swiss-STR. Relevant information, not limited to spinal lesions, but encompassing oncological details, surgical intervention, overall performance status, and QoL will be collected in accordance with the pertinent literature on spinal oncology. The registry will provide referring caregivers and healthcare professionals with a valuable tool to standardize, assess, and compare current treatments in spinal cancer patients. Moreover, the Swiss-STR will serve as a catalyst in the development of evidence-based treatment algorithms, foster scientific collaboration among centers, and address a number of remaining open questions in the management of tumors affecting the spine.

While primary spine tumors are treated in a few, highly specialized centers, metastatic lesions to the spine are treated at many institutions that offer complex spine surgery. Recent progress in defining key parameters necessary for clear patient description has significantly facilitated communication in the field of spinal oncology (5). However, if surgery is indicated, many treatment options might be offered for similar conditions and the final management strategy for an oncological spine disorder is often decided by the spine surgeon. This scenario makes it difficult to perform a randomized cohort study of surgical techniques (62). The registry will overcome this limitation by a prospective collection consisting of large-scale, high-quality data from which the effect of confounding variables can be further adjusted. The registry will conduct primary descriptive studies in surgically treated spinal tumors and assess the effectiveness of interventions. Unbiased, continuous, and robust patient data permits monitoring of the socio-economic burden of this healthcare condition and assessing of clinical outcomes.

The heterogeneity of tumors that affect the spine and the multimodal, interdisciplinary approach in patient care presupposes precision medicine in the implementation of a purposeful oncological treatment. Molecular cancer-tissue analysis has played a role in changing the treatment paradigm: the more stochastic “one-size-fits-all” cytotoxic treatment approach is transforming toward precise assessment and identification of tumor-specific vulnerabilities to define potential drug targets.

Modern systemic treatment modalities attack cancer cells through two primary methods: (1) pathway-based targeted therapy selectively disrupts pathways necessary for cancer cell survival or growth, while (2) immunotherapy artificially modulates a patient's immune system to generate a response against cancer cells (63). However, a major limitation of targeted anticancer therapies is intrinsic or acquired drug resistance. Molecular disease monitoring represents a logical way forward to delay and ultimately overcome the development of drug resistance (64). The establishment of a biobank allows for insight into tumor biology. Subsequently, genetic, transcriptional, and proteomic analyses becomes feasible and possibly patient-derived *in vitro* cell models. With the available clinical data, association of genomic features with clinical information can be defined and thereby outline the importance of a simultaneous comprehensive clinical registry.

Since the initial launch of case-based cancer registries in the early 1900s (65), registries have expanded globally to yield robust evidence in healthcare research (66). Generally, three types of cancer registries exist. First, facility-based registries collect information about patients treated in that institution. Second, specialty-based registries collect data about one specific type of medical condition. Third, central registries collect information about patients in a certain geographic area (65, 67). Notably, our specialty-based registry aims to provide interested institutions with a comprehensive data assessment tool while facilitating data pooling and scientific collaboration. Although different international registries are available on spinal tumors, the Swiss-STR creates a tailored prospective-data-capture platform to facilitate fast and robust data transfer from common patient electronic charts.

## Conclusion

Swiss-STR is an important step in documenting treatment and outcome of a complex disease that affects a broad population suffering from cancer. Furthermore, this registry will help define its socio-economic burden and reflect what is currently believed to be the best medical treatment or standard of care. Finally, the registry will help to foster research between institutions and thereby further push the envelope in spinal oncology research.
