# Supplementary material for: Establishing the Swiss Spinal Tumor Registry (Swiss-STR): a prospective observation of surgical treatment patterns and long-term outcomes in patients with primary and metastatic spinal tumors
Source: Front Surg. 2023 Jul 28;10:1222595. doi: 10.3389/fsurg.2023.1222595 (PMC10416635; doi:10.3389/fsurg.2023.1222595)
Supplement: Supplementary file 1 [file Table1.docx]

Supplement Table 1: Data collection schema

|  | | | | | | | |
| --- | --- | --- | --- | --- | --- | --- | --- |
| **Preoperative visit** | **Surgery** | **6 weeks visit** | **3 months visit** | **6 months visit** | **12 months visit** | **24 months visit** | **Extra visits for** |
|  | | | | | | | |
| Basic *demographic* characteristics | *Date of surgery* | Basic *demographic* characteristics | Basic *demographic* characteristics | Basic *demographic* characteristics | Basic demographic characteristics | Basic demographic characteristics | Ad on surgeries |
| *Comorbidities* | *Operation time* | *Comorbidities* | *Comorbidities* | *Comorbidities* | *Comorbidities* | *Comorbidities* | Emergency consultation |
| Current *Medication* including pain killers and chemotherapy | *Surgeons* involved   - Neurosurgeons - Orthopedic | Current *Medication* including pain killers and chemotherapy | Current *Medication* including pain killers and chemotherapy | Current *Medication* including pain killers and chemotherapy | Current *Medication* including pain killers and chemotherapy | Current M*edication* including pain killers and chemotherapy | Recurrent tumor |
| Primary tumor *TNM status* | *Surgical treatment*   - Biopsy - Separation surgery - Spondylectomy - Thermoablation | *Histopathology* of spinal lesion | *Radiation oncology* treatment |  |  |  | Additional visits |
| Basic *laboratory values* including tumor markers | *Surgical access*   - Open surgery - Minimally invasive surgery | Basic *laboratory values* including tumor markers | Basic *laboratory values* including tumor markers | Basic *laboratory values* including tumor markers | Basic *laboratory values* including tumor markers | Basic *laboratory values* including tumor markers |  |
| *Clinical performance*   - Neurological assessment - ASIA score - Timed-up and go test - Karnofsky index | *Instrumentation* used   - Screws/rods - Interbody devices - Cement augmentation | *Clinical performance*   - Neurological assessment - ASIA score - Timed-up and go test - Karnofsky index | *Clinical performance*   - Neurological assessment - ASIA score - Timed-up and go test - Karnofsky index | *Clinical performance*   - Neurological assessment - ASIA score - Timed-up and go test - Karnofsky index | *Clinical performance*   - Neurological assessment - ASIA score - Timed-up and go test - Karnofsky index | *Clinical performance*   - Neurological assessment - ASIA score - Timed-up and go test - Karnofsky index |  |
| *Radiological assessment*   - Spinal instability neoplastic score (SINS) - Epidural spinal cord compression scale (ESCC) - Sarcopenia Index | Intraoperative *adverse events* | *Radiological assessment*   - X-ray in case of instrumentation | *Radiological assessment*   - X-ray in case of instrumentation | *Radiological assessment*   - X-ray in case of instrumentation | *Radiological assessment*   - X-ray in case of instrumentation | *Radiological assessment*   - X-ray in case of instrumentation |  |
| *Health-related quality of life*   - Spine Oncology Study Group Outcomes Questionnaire 2.0 - EQ-5D-5L - Oswestry Disability Index (ODI) including numeric rating scale (NRS) - Neck Disability Index (NDI) including numeric rating scale (NRS) | *Intraoperative monitoring*   - Neuromonitoring - Tumor-neural separation assessment using intraoperative ultrasound | *Health-related quality of life*   - Spine Oncology Study Group Outcomes Questionnaire 2.0 - EQ-5D-5L - Oswestry Disability Index (ODI) including numeric rating scale (NRS) - Neck Disability Index (NDI) including numeric rating scale (NRS) | *Health-related quality of life*   - Spine Oncology Study Group Outcomes Questionnaire 2.0 - EQ-5D-5L - Oswestry Disability Index (ODI) including numeric rating scale (NRS) - Neck Disability Index (NDI) including numeric rating scale (NRS) | *Health-related quality of life*   - Spine Oncology Study Group Outcomes Questionnaire 2.0 - EQ-5D-5L - Oswestry Disability Index (ODI) including numeric rating scale (NRS) - Neck Disability Index (NDI) including numeric rating scale (NRS) | *Health-related quality of life*   - Spine Oncology Study Group Outcomes Questionnaire 2.0 - EQ-5D-5L - Oswestry Disability Index (ODI) including numeric rating scale (NRS) - Neck Disability Index (NDI) including numeric rating scale (NRS) | *Health-related quality of life*   - Spine Oncology Study Group Outcomes Questionnaire 2.0 - EQ-5D-5L - Oswestry Disability Index (ODI) including numeric rating scale (NRS) - Neck Disability Index (NDI) including numeric rating scale (NRS) |  |
|  | | | | | | | |
